# Limit values for the web instrument Need for Recovery based on health over two years and corresponding psychosocial working environment

**DOI:** 10.1177/10519815241289830

**Published:** 2024-12-14

**Authors:** Kerstin AH Wentz, Mats Hagberg

**Affiliations:** 1 Occupational & Environmental Medicine, Sahlgrenska University Hospital, Gothenburg, Sweden; 2 Occupational & Environmental Medicine, University of Gothenburg (UGOT), Gothenburg, Sweden

**Keywords:** occupational stress, mental fatigue, weights and measures, calibration, internet-Based intervention, rehabilitation, health surveys, public health surveillance

## Abstract

**Background:**

The magnitude of fatigue or need for recovery (NFR) from work shifts could be used as a tool for health surveillance and for monitoring rehabilitation.

**Objective:**

To develop a model to calculate the healthy limit value for NFR based on health over two years in a cohort of workers and from there healthy limit values for the psychosocial work environment. The model is to be used in a web instrument.

**Methods:**

Based on survey data from 1289 engineers, carpenters, nurses and home care employees, NFR was calibrated against six Copenhagen Psychosocial Questionnaire (COPSOQ II) health scales. Regression models explored the correspondence between NFR at baseline and regularity in function impact on health over two years. Thereafter, the limit values for the psychosocial work environment were examined. Successive calculations concerned sensitivity and specificity of these limit values.

**Results:**

The baseline NFR value that predicted a set minimum level of regularity of impact on health from each health scale two years later (p < 0.001), formed a mean minimum of regularity NFR score of = 9.02. The limit healthy NFR value was set below this value to 9/33 points. Sensitivity regarding the limit healthy NFR value concerning prediction of an unhealthy NFR was 85%. Specificity regarding healthy cases was 74%. Healthy limit values for the psychosocial work environment concerned first of all work process resources and then work demands.

**Conclusions:**

A Need for Recovery limit value could be based on the frequency of the fatigue reaction and function impact on health (frequency).

## Introduction

The importance of the magnitude of fatigue or need for recovery (NFR) after work shifts is about well-being in everyday life^1^ and about the ability to create a state of recovery after work.^2^ In addition, the magnitude of the post-workload reaction also could predict serious disease.^3^

Therefore, we started from the Dutch Need for Recovery Scale (dutchNFR),^4^ in order to develop the web instrument Need for Recovery from work (webNFR) http://skattaaterhamtning.se/en.^5^ The webNFR, which is also available in English, is designed for single employees but could also be used by groups of employees. It could further be used by the occupational health services as a tool for surveillance of health^4^ or to tailor and progressively monitor employee rehabilitation to prevent relapse into ill health. These different uses require a healthy limit value for NFR.

In order to create a model for calculating the limit values for the Swedish Need for Recovery Scale (SwedishNFR) to be used in the webNFR, we reviewed the state of knowledge necessary to calculate the limit value for fatigue at the end of work shifts and from there also derived limit values for the psychosocial working environment.

### Empirical and theoretical background for calculating limit values for NFR and exposures at work

#### Stress, the time for recovery and NFR

Challenges or stressors are present in all spheres of life.^6^ When based on the work process, challenges can be distinguished from stress when previously acceptable challenges at work become excessive. In this situation the work demands are not matched to the individual's knowledge, abilities, and ability to cope.^7^ Moreover, stressors can also emerge from a poor work design, such as insufficient resources to do the job, lack of autonomy or control, or insufficient support from colleagues or managers.^7^ As a consequence, work stress can damage an employee's performance^8^ and health^7^ as well as the business performance.^7^

Most workers need to cope with exposure to many stressors and different stressors at the same workplace. This makes high-quality quantification of job stress and surveillance of stress and health a challenge; however, a good measure of both the severity of load from the work process and future health effects is the time required to recover from the different stressors.^3^ At the same time, the mediating and readable load response NFR lies between the work stress and the recovery process (at the end of shifts)^3,9^ and represents the time for recovery from stressors.^3^ The significance of this load reaction is also emphasized by fatigue during the day reaching its highest value at this time.^10,11^ Furthermore, the individual's measured value of NFR corresponds to the values of fatigue at all other times (e.g., morning before work, during work,^10,11^ or after the weekend/vacation^11^).

When the recovery process is prolonged, the employee will begin an upcoming work shift in a suboptimal condition that also requires compensatory effort to do the job. In turn, this brings increased load reactions, and through accumulation a risk for more chronic load reactions^12^ and a more chronic state of fatigue,^4^ while NFR should be understood as a short-term load reaction that is found to be different from exhaustion per the Maslach Burnout Inventory.^13^

#### NFR, workload, and physiological state

Researchers agree on that a certain amount of NFR from work is expected,^10,14^ by which is meant a short-term load reaction.

Cross-sectional NFR relates to working conditions such as mental and emotional work demands, the length of working hours,^15,16^ or lack of control over breaks.^1,16^

Longitudinally, high work demands or low support from colleagues meant a high risk for a high NFR six months later.^17^ Similarly, an earlier increase in working hours among nurses resulted in increased NFR.^18^ Conversely, rescheduling of shifts to increase time for recovery between shifts concerning coach drivers (less residual fatigue when working) meant a 50% analogue reduction in NFR, general fatigue, emotional exhaustion, and so-called psychosomatic complaints.^19^

Self-reports on work stressors have repeatedly been confirmed by autonomic nervous system imbalance *and hypothalamic-pituitary-adrenal axis regulation disparities also including cortisol dynamics* (stress hormones).^20^ This type of imbalance has in part been explained by work demands, where the NFR measurement was also corroborated by an increase in adrenaline at a baseline level (day off) and a delayed adrenaline recovery during leisure time. NFR was further related to increased cortisol reactivity during work and to delayed cortisol recovery after work.^15^ Similarly, Elfering et al.^14^ reported that observers’ rated higher workload entailed delayed physiological recovery (increased levels of cortisol) during a second day off from work. The association between different levels of workload and regulation of cortisol was fully mediated by the NFR of the employees.^14^

#### NFR is one of several impacted functions

The psychometric instrument that measures NFR captures a load reaction from work that consists of impact on functions that, besides fatigue, means a need to withdraw socially, difficulties concentrating, and delayed recovery.^4^ Other forms of impact on functions, so-called psychosomatic complaints, headaches, or muscle pains, are first predicted by NFR,^16^ and second, in the same way as NFR, predicted by cortisol and adrenaline measurements. While NFR is related to length of working hours,^15,16^ there are also additional recorded impacts such as less positive affect, more negative affect, and increased blood pressure.^21^ It is noteworthy that multiple recorded forms of failing functions consistently appear together,^1,22^ including so-called psychosomatic complaints that show a psychometrically high level of homogeneity concerning manifestations such as shortness of breath, headaches, easily upset stomach, dizziness, fatigue, and so forth.^23^

#### Fatigue as an impact on functions impedes switching off from work

Fatigue impedes functions such as mobilization of energy,^24^ and when working, this also means that load from compensatory effort is added to the load from the work demands themselves.^25^ In addition, for the employee to decide to continue working despite being tired,^26^ this decision also increases the cognitive load from self-control, such as attention to work tasks ahead and resisting the need to rest.^26^ Because this self-control is also regulated by the remaining available cognitive regulatory capability,^26^ and both fatigue and self-control tax this cognitive capability, this will also negatively impact the cognitive ability to switch off from work to achieve a state of recovery. This negative effect on the ability to recover has been reported from both increased workload^2,27^ and, as expected, also by the magnitude of NFR.^2^

### The cut-off value from the frequency of the load reaction

#### Frequent or infrequent NFR

Stable high levels of work demands meant over 12 months a step-by-step increase in global fatigue,^28^ which is equivalent to a reduced ability to recover. A more profound transformation, as from a high (frequent) NFR to exhaustion, has also been suggested as a failing ability to recover, despite being on sick leave.^29^

Instead, infrequent exposure to stress appears to allow for recurrent well-being, where the inability to unwind after work^2^ and disturbed sleep^2,27^ is more limited to days with increased work demands. When work demands are balanced, this seems to correspond to a frequency of NFR in the middle between never and sometimes, and impact on functions being episodic.^1^

The extent of fatigue is typically registered via the frequency of the fatigue reaction^4,10,11^; from the perspective of quality of life and health it is crucial that a short-term load reaction NFR appears, if at all, only sparsely and thereby not cumulatively.

#### Prediction of future impact on functions and risk for health

Impact on functions such as fatigue and difficulties concentrating can be regular^28^ or irregular.^27^ A high NFR from work was found to mean a psychometric score of approximately often. Infrequent NFR from work means a score centred in the middle between never and sometimes. Both infrequent and frequent NFR are in proportion to the occurrence of impact on functions,^1^ including general fatigue.^10,11^ While NFR relates to the quality of physiological regulation,^14,15^ a heightened NFR also means a prospective risk for long-term health effects such as cardiovascular disease over 32 months^3^ or longer sick listing.^30^ Logically, general measures of fatigue are also prospectively related to longer sick leave, and to an even greater degree than disturbed sleep.^24^ Furthermore, Barlas^31^ documented how fatigue in terms of health-related quality of life (SF-36) could predict stroke in the general population. In addition, excessive fatigue has been shown to predict myocardial infarction.^32^

#### Limit values for NFR from work from prediction of health

A limit value for NFR aims at current and future quality of life or impact on functions, including physiological dysregulation and risks concerning health and well-being.

A web instrument that records the current level of health should be able to predict future health and well-being based on data from longitudinal survey measurements dealing with future health and well-being of not experiencing regular impact, on the Swedish version of the Copenhagen Psychosocial Questionnaire (COPSOQ) II) scales for health and well-being.^33^ This healthy limit value for NFR needs to be supplemented with limit values for those psychosocial working conditions that longitudinally often have the greatest significance for (i.e., can predict) the level of NFR.

### Aim

The aim was to develop a model for calculating the limit values for the webNFR, based on health during two years for a study group of workers. Based on the limit value for NFR from work, the webNFR could capture both meaningfully reduced quality of life and physiological dysregulation before serious disease. Starting from the state of knowledge, the limit value of the instrument NFR should be based on and calibrated from impact on functions, for example, impaired sleep and cognitive functioning that are not regular but only sparsely recurring. The corresponding limit values concerning the psychosocial working environment that do not jeopardize a healthy NFR should be examined cross-sectionally and longitudinally. In addition, the validity of the webNFR in terms of sensitivity and specificity of all limit values should be examined as well.

## Methods

### Procedure and participants

The selection of respondents was based on age and occupations with differential exposure to physical load, mental load, or physical and mental load. A further alignment was to include professions with high versus low rates of occupational injuries, where architects and engineers represent a clearly low rate of reported occupational injury.^34^ The occupational groups were architects and engineers with a five-year university degree, carpenters working at building sites, registered nurses working in hospitals, and home care employees working in the homes of care recipients. Random sampling was done based on the Swedish Standard Classification of Occupations (SSYK), using the above criteria and including individuals 19–70 years of age. Sampling was done using proximity sampling in the Västra Götaland Region or within a specified municipality in Västra Götaland Region.

In a first step, Statistics Sweden sent letters of invitation to 1250 representatives in each of the four occupational groups. The invited individuals were requested to mark “other” if their current occupational group had changed. The letter also included a numbered consent form, the survey, and a return envelope. For detail on procedure, see publication based on baseline measurement data.^1^ A total of 1292 out of 5000 individuals responded and were included in the study. At baseline the response rate from the occupational groups was 18.0%, 12.5%, 43.4%, and 8.6% for architects and engineers, carpenters, hospital nurses, and homecare employees, respectively. This total sample at baseline had an NFR mean score of 11.6 points (SD 6.0).^1^

**Figure 1.**
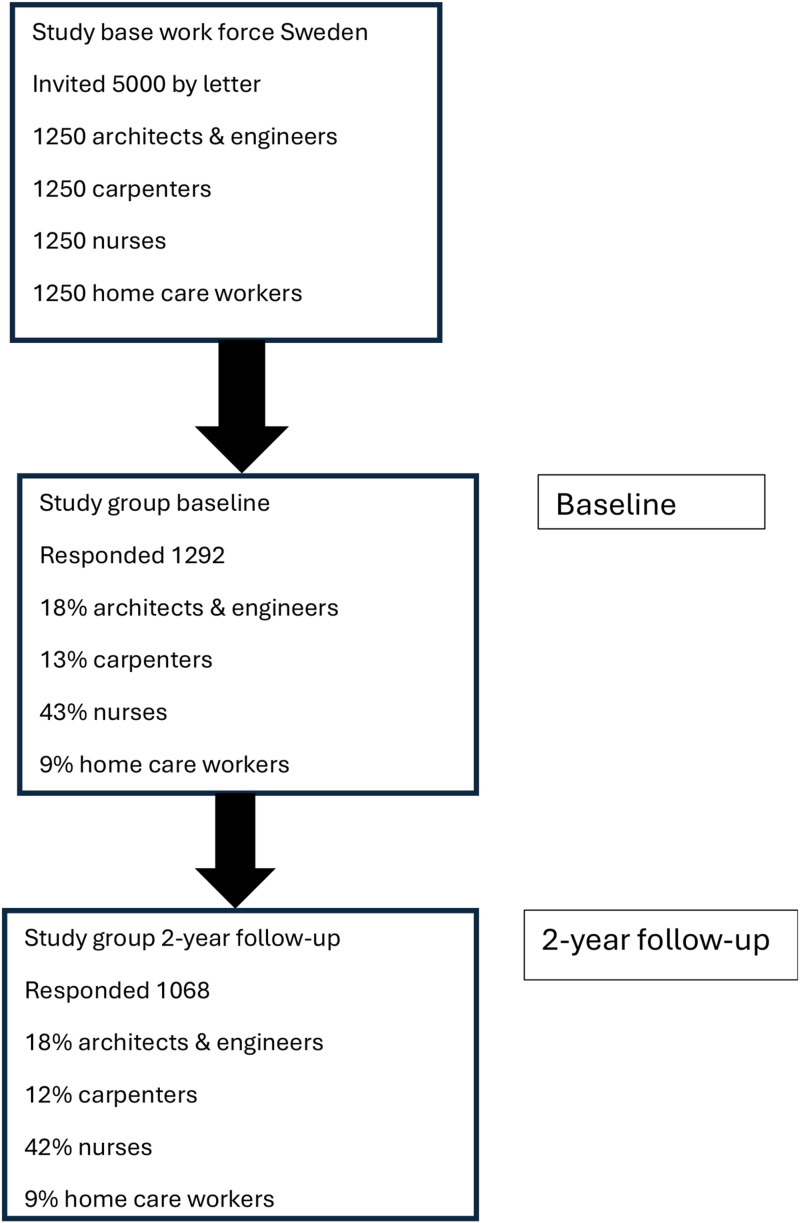
Flow chart of participants in the study.

At the two-year follow-up there was an 83% response rate (1068 participants) with a current mean NFR score of 11.1 points (SD 6.2) (1015 respondents). Two years earlier this subsample had presented a mean NFR score of 11.4 points (SD 6.0). At baseline this subsample was composed of 17.9%, 12.2%, 42.3%, 9.0%, and 18.6% architects and engineers, carpenters, hospital nurses, home care employees, and other, respectively. After two years the sample was composed of 18.4%, 11.3%, 44.6%, 7.3%, and 18.3%, in the same order. (Figure 1)

### Instruments and variable

The total survey consisted of 115 questions. The questionnaire contained items on background data: age, gender, family situation and employment, sick leave, and exercise habits, followed by items covering psychological health and well-being, including NFR*.* In order, further items concerned physical complaints, physical demands, exposure to vibrating tools, compensating (psychological) strategies for managing work, and psychosocial work environment factors. Data on sick leave, health behaviors including strategies for doing the work, physical demands, and exposure to vibrating tools were not included in the present analysis. Concerning background variables and recovery profiles for the different occupations, see.^1^

#### Work demand scales from COPSOQ II

The Swedish version of the COPSOQ II medium-size questionnaire, adapted by Berthelsen et al.,^33^ is an instrument that comprises the work demand scales Quantitative demands (four items, e.g., *“*Do you fall behind in your work?”), Work pace (three items, e.g., “Is it necessary to keep working at a high pace?”), and Emotional demands (three items, e.g., “Is your work emotionally demanding?”). The COPSOQ II long version for research use Cognitive demands scale (four items, e.g., “Does your work require you to remember a lot?”) was adapted by the research group (see section on health scales).^1^ The items of two scales, Work pace and Emotional demands, have five response options ranging from “To a very small degree” to “To a very high degree.” The other COPSOQ II scales have five response options ranging from “Never/almost never” to “Always.” The response options for all items of the six scales were translated into 0, 25, 50, 75, and 100 points. Each scale has a total score ranging from 0 to 100 points based on the item average. Higher scores indicate a larger number of demands or resources. For details regarding these items, see Berthelsen et al.^33^

#### Work process resources scales and items

Some of the work process resources scales were collected from the Swedish version of COPSOQ II (see paragraph on demands). These were Influence (three items, e.g., “Can you influence what you do in your work?”), Social support from colleagues (three items, e.g., “How often do you get help and support from your colleagues?”), and Social support from the supervisor (three items, e.g., “How often do you get help and support from your nearest superior?”). Concerning response options and translation into points, see the previous subsection.

Aronsson et al.^11^ used single items to examine work process resources. One of these concerned Influence over resources when doing work: “Are you and your work group able to influence how many resources you will have for carrying out your work?” Another concerned Time to reflect: “Does your work mean that there is time for reflection and discussions about working methods and experiences at work?” Four items reflected the opportunity to deliver good Quality of work (e.g., “Do you have adequate resources to perform your work in a way that is satisfactory to you?”). These items had five response options ranging from Never = 0 to Very often/always = 4. The single items of Quality of work composed a scale termed Quality of work. A good internal consistency was found for the scale (Cronbach's alpha = 0.795) among 1280 participants.^1^

The work process resources scale Recovery opportunities records degrees of freedom belonging to the work shifts, for example, breaks and scheduling of shifts. The Recovery opportunities scale was adapted from Dutch^35^ to Swedish conditions in accordance with the guidelines for cross-cultural adaptation of self-report measures on health proposed by Beaton et al.^36^ and tested in Swedish samples.^37^ The scale comprises nine items such as “Can you decide yourself when you take a break?” The items have four response options ranging from 0 = “Never” to 3 = “Always”. Higher scores indicate greater opportunities for recovery. Good internal consistency was found for the Swedish version of the Recovery opportunities scale (Cronbach's alpha = 0.803) among 142 participants.^37^

#### NFR and additional health and well-being scales

The 11-item SwedishNFR records cumulative load effects centered on the end of the working day through items like, “I cannot really show any interest in other people when I have just come home myself” and “By the end of the working day I feel really worn out.” The scale^4^ was adapted from the DutchNFR to Swedish conditions in accordance with the guidelines for cross-cultural adaptation of self-report measures on health, proposed by Beaton et al..^36^ For the scale to be used for individual psychometrics, the adaptation process included a transition to four-point response options in accordance with van Veldhoven.^38^ For the adaptation process, see.^37^

Further health scales were recorded using the Health and Well-being scales from the Swedish version of the COPSOQ II^33^ or by the research group using the cross-cultural adaptation process^36^ from the English version of COPSOQ II long version for research use. The scales adapted by Berthelsen et al.^33^ record cognitive and emotional expressions of Stress, by using items such as “How often have you felt stressed?”, Problems sleeping by asking, “How often have you slept badly and restlessly?”, and Burnout by using items like, “How often have you felt emotionally exhausted?” The scales adapted by the research group and tested in Swedish samples^1^ record Depression, for example, by asking, *“*How often have you lacked self-confidence?”, Cognitive stress by asking, “How often have you had difficulty remembering”, and Somatic stress using items such as “How often have you had heart palpitations?” The five response options range from 0 = “Never/almost never” to 100 = “All of the time” and concern the last four weeks. Higher scores indicate worse health.

### Statistical analysis

#### NFR limit values from longitudinal prediction of health

We used baseline data and made a cross sectional prediction from NRF concerning each of the six health scales to be used in a web instrument prototype.

**Figure 2.**
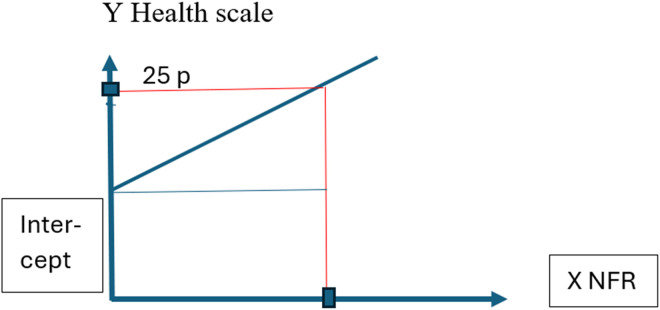
An example of the composition of six regression models where the regression lines are based on the Y-axis determined by the values of the unstandardized constants B, the intercept. The unstandardized regression coefficient B represented steps on the Y axis corresponding to one step on the NFR scale.

The healthy longitudinal limit value for NFR was calculated from participants who completed the survey again after two years. First, a limit healthy NFR value was set to the level of NFR that predicted impact on health two years later that was below the minimum of regularity in impact on health. Second, we created six regression models with baseline NFR as the predictor and each health measurement two years later as dependent variables. Concerning the health scales Stress, Sleep problems, Burnout, Somatic stress, Depression and Cognitive stress, from COPSOQ II,^1,33^ the minimum of regularity in impact on health was the response option “A little bit of the time,” that is, 25 points*.* Third, the correspondence between the NFR scale steps and 25 health scale steps, was inferred from the regression lines of the six regression models. These regression lines were formed from the unstandardized constant B, that is, where the regression line is based on the y-axis (the intercept) as shown in Figure 2. Thereafter the unstandardized regression coefficient (B) (that represents the scale steps on the Y axis corresponding to one step on the NFR scale) was extracted (Figure 2).

Fourth, these two coefficients were inserted into a mathematical expression derived from the straight-line equation Y = a + bX, which also can be written as X = Y – a / b,^39^ where the NFR is represented on the x -axis. This equation reads NFRminimum regularity = (25 – intercept) / B. Fifth, the minimum recorded level of regularity of impact on health two years later was calculated from the mean of the NFR scores, namely, X_1_ + X_2_ + X_3_ + X_4_ + X_5_ + X_6_ / 6. Sixth, the limit value for a healthy NFR was set to the full NFR scale step below this minimum regular impact on health two years from baseline. Seventh, from the regression models we extracted also the standard error of the unstandardized constant B and the unstandardized regression coefficient (B) together with the 95% confidence interval for each coefficient.

#### The limit healthy NFR value sensitivity and specificity

Instrument sensitivity and specificity^40^ refers to predictive reliability of the limit healthy NFR value to correctly classify unhealthy or healthy NFR cases two years after the predictive baseline measurement. Sensitivity was calculated from the comparison between correctly identified unhealthy cases at baseline and the sum of correctly classified as unhealthy and from baseline shown to be incorrectly classified healthy cases. In the same way, specificity means comparison between correctly classified healthy non-cases from baseline and the sum of correctly and incorrectly predicted (as unhealthy) healthy non-cases at follow up.

#### Working conditions that best explain NFR

Which four dimensions of working conditions could best explain NFR after two years was examined gradually through stepwise regression analysis concerning (1) the effect from psychosocial working conditions at baseline on concurrent NFR, (2) the effect from baseline psychosocial working conditions on NFR two years later, and (3) the impact from the concurrent psychosocial working conditions on NFR after two years. Thereafter, the result from each of these four psychosocial working environment predictors of NFR were divided into the healthy score (0 to limit healthy NFR) subsample and the unhealthy score ((limit healthy NFR + 1) to 33) subsample. Concerning these subsamples, a normal distribution was approximated; healthy working conditions were delimited based on he means of the healthy subsample plus or minus 0.5 SD covering approximately 70% of the distribution (concerning) each of the four different psychosocial working environment scores. The corresponding calculations were also applied to the unhealthy subsample.

#### Sensitivity and specificity of the NFR limit value for a healthy working environment

We examined the sensitivity of the limit healthy NFR value to correctly classify cases in terms of unhealthy levels of demands and resources at work and the specificity of the limit healthy NFR value classifying concurrent healthy levels of demands and resources at work.

## Results

### The limit value of the health scale NFR over two years

A healthy limit value of NFR was calculated progressively from 1042–1047 participants (see Table 1).

**Table 1.** Six models with each of COPSOQ II health scales stress, sleep problems, burnout, somatic stress, depression, and cognitive stress as the dependent variables, and NFR as the predictor. Unstandardized coefficients B (scale steps) and 95% confidence intervals of the unstandardized coefficients B for each model.

Model				
Constant	Unstandardized coefficient B	Coefficient standard error	95% confidence interval for B	N
NFR				
1. Stress	11.23	1.151	8.97–13.491	1042
NFR	1.81	0.089	1.6–1.99	1042
2. Sleep problems	13.91	1.180	11.80–16.23	1043
NFR	1.17	0.91	0.99–1.35	1043
3. Somatic stress	7.98	1.057	5.9–10.06	1042
NFR	1.39	0.082	1.23–1.55	1042
4. Burnout	11.29	1.103	9.13–13.45	1047
NFR	1.06	0.086	1,80–2.12	1047
5. Depression	8.86	1.159	6.59–11.13	1042
NFR	1.78	0.090	1.60–1.96	1042
6. Cognitive stress	9.24	1.162	7.00–11.52	1042
NFR	1.80	0.090	1.63–1.98	1042

Table 1 shows six regression models of prediction from NFR at baseline of Stress, Sleep problems, Burnout, Somatic stress, Depression, and Cognitive stress two years later. Each regression model is presented in terms of unstandardized coefficients (scale steps) where the health scales coefficient B represent in scale steps where the regression line is based on the y-axis. The NFR B coefficient means the proportion in scale steps on the y-axis to one (NFR) step on the x-axis.

**Figure 3.**
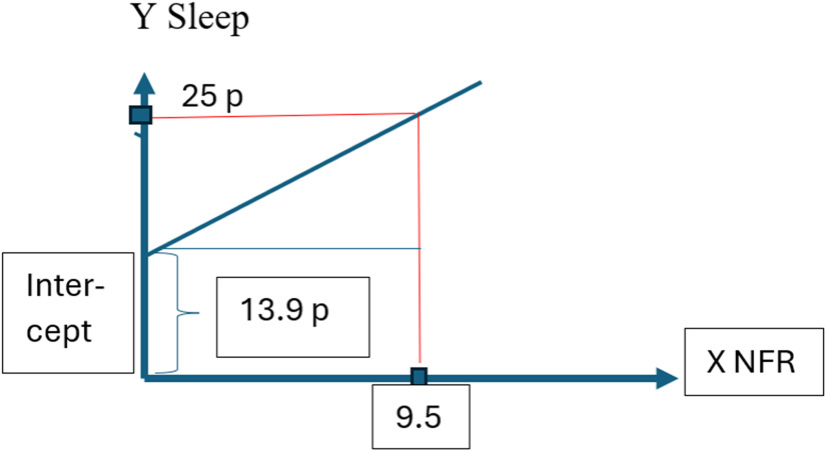
The extracted coefficients in scale steps for the health scale sleep and in scale steps for NFR means a calibration of NFR to the value of minimum of regularity of impact on health concerning sleep. This value concerning the Sleep scale is 25 points. The calibration of the NFR scale results in 9.5 NFR scale steps.

All six regressions models showed significant associations (p < 0.001) between NFR at baseline and a minimum level of regular health impact concerning each health measure at baseline or two years later. Figure 3 gives an example of calibration of NFR from a health scale, Sleep, with a known value of a minimum of regularity of impact on health of 25 points. The NFR scale calibration with this health scale means 9.5 NFR scale steps.

The NFR score that corresponded to the minimum of registrable regularity of impact on health, 25 points, was, as seen in Table 2 in terms of Stress, 7.6 points; Somatic stress, 12.2 points; Sleep problems, 9.5 points; Burnout, 7.0; Depression, 9.1 points; and Cognitive stress, 8.7 points. The mean NFR point correspondence to 25 health scales points was 9.02. The limit healthy NFR value of less than minimum regular impact on health after two years was in full scale steps 9.0 (of a maximum of 33 points).

**Table 2.** Baseline *need for recovery* (NFR) scores that corresponded to 25 points “a little bit of the time” on the COPSOQ health scales after two years. Baseline cross sectional correspondence between NFR and 25 points on the COPSOQ health scales not used in the present analysis.

	Stress	Somatic stress	Sleep problems	Burnout	Depression	Cognitive stress	Mean score
Over 2 years N = 1042–1047	7.6	12.2	9.5	7.0	9.1	8.7	9.02
Baseline N = 1258–1263	7.6	11.8	10.3	7.6	9.2	9.0	9.3

Table 2 shows the need-for-recovery (NFR) scores at baseline and two years later that corresponded to the minimum of regularity of impact on health: 25 points on the COPSOQ II health scales regarding Stress, Sleep problems, Burnout, Somatic stress, Depression, and Cognitive stress*.* Corresponding cross-sectional results from baseline are presented in brackets.

### Sensitivity and specificity of the limit value

Sensitivity predicting an unhealthy NFR from baseline as the ratio between 474 cases correctly classified as unhealthy two years later and the sum of 474 cases correctly classified and 84 falsely classified as non-cases two years later: 474 / (474 + 84) = 0.849.

Specificity predicting a healthy NFR as the ratio between the 338 non-cases correctly classified from baseline and the sum of these 338 non-cases and falsely classified 119 non-cases two years later: 338 / (338 + 119) = 0.739.

Sensitivity was 85% and specificity was 74%.

### The importance of different working conditions for NFR

The importance of the different psychosocial working conditions for NFR at baseline and two years later was examined using stepwise linear regression analysis. Table 2 displays how the baseline predictors of the demands and resources for doing the job explained 39% of the variance in NFR at baseline and 27.1% of the variance in NFR two years later. At this later occasion, the concurrent demands and resources for doing the job best explained the variance in NFR, 40.2% (see Table 3). The two most important predictors in all three models were Quality of work and Recovery opportunities, which both showed negative values of R^2^. This means that these resource scales contributed to the variance in NFR by decreasing the NFR from work.

**Table 3.** Stepwise linear regression analyses with *need for recovery* (NFR) at baseline and after two years as the dependent variables and baseline psychosocial working conditions or psychosocial working conditions after two years that reached statistical significance at each occasion. Potential predictors were Quality of work, Recovery opportunities, Quantitative demands, Emotional demands, Work pace, Social support from colleagues, Cognitive demands, Influence, Influence over resources, Time to reflect, and Social support from manager.

Predictor variables Beta P-value	Quality of work	Recovery opportunities	Quantitative demands	Emotional demands	Social support colleagues	Work pace	Model summary	Total adjusted R	F of the regression equation	Significance of F
Baseline working conditions predicting concurrent need for recovery N = 1212	−0.301 <.001	−0.221 <.001	0.119 <.001	0.101 <.001	−0.076 <.001	0.121 <.001		0.390	130.359	<.001
Baseline working conditions predicting need for recovery 2 years later N = 982	−0.324 <.001	−0.173 <.001		0.110 <.001	−0.058 <.001	0.071 <.001		0.271	74.230	<.001
Working conditions after 2 years predicting concurrent need for recovery N = 953	−0.298 <.001	−0.216 <.001	0.124 <.001	0.145 <.001	−0.082 <.001	0.080 <.001		0.402	108.602	<.001

The result shown in Table 3 is based on data from four different professions and an occupational group labeled “other”.^1^ At baseline there were 1212 participants, and two years later there were data from 982 participants from the same sample. On both occasions, the significant predictor variables were Quality of work, Recovery opportunities, Quantitative demands, Emotional demands, Social support from colleagues, and Work pace. In both models the variables Cognitive demands, Influence, Influence over resources, Time to reflect, and Social support from manager were not significant predictors. The predictors that best explained the variance in NFR after two years (third model) were the concurrent predictors. Their validity could be examined from another data set,^41^ in this case baseline data. The results were common to both models, which supported the use of these predictors.

### A limit healthy NFR and limit values for work demands and resources

Limit values after two years regarding the resources Quality of work and Recovery opportunities were calculated from the concurrent impact on functions of NFR with a limit healthy NFR value of 9 points.

The calculation was based on an analysis of the data from the measurement after two years, which showed that 45.2% of the participants reported a limit healthy NFR score within the range 0–9 points. In the limit healthy NFR 0–9 points subgroup (showing beneficial health measurements scores) the mean score on the Quality of work scale was 12.4 points and on the Recovery opportunities scale was 17.8 points. The limit value for these conditions was set to 0.5 SD below the mean. Thus, the limit value for the scale Quality of work became 11 points and for the scale Recovery opportunities, 15 points.

Concerning Quantitative demands and Emotional demands, one single item was selected from each scale. These items were “Do you have enough time for your work tasks?” and “Is your work emotionally demanding?” The user was asked to answer these questions with Yes or No.

The estimated cut-off values regarding the resources Recovery opportunities and Quality of work based on the limit healthy NFR value of 9 points and the single items of Quantitative demands and Emotional demands were examined from the perspective of sensitivity of an unhealthy NFR score to unfavorable levels of demands and resources at work.

The sensitivity of concurrent delimiting working conditions, which belongs to an unhealthy NFR two years from baseline, was calculated as a ratio of 329 unhealthy NFR cases correctly classified at an unhealthy (low) level of access to Recovery opportunities to the sum of these 329 cases and 130 falsely classified as healthy concerning Recovery opportunities. This reads as 329 / (329 + 130) = 0.716. Concerning the experience of Quality of work, the corresponding calculation reads as 338 / (338 + 111) = 0.752.

The specificity of the limit healthy NFR concerning access to a healthy level of Recovery opportunities was examined, where 294 healthy NFR cases were correctly classified as having healthy (higher) access to Recovery opportunities, and 250 were falsely classified at an unhealthy (low) level of access to Recovery opportunities: 294 / (294 + 250) = 0.540. Concerning the experience of Quality of work, the corresponding calculation was 313 / (313 + 261) = 0.545.

The original response options of the single items Quantitative demands and Emotional demands were classified into Yes or No categories. (Never/hardly ever, Seldom = No; To a very small extent, To a small extent = No; Sometimes, Often, Always = Yes; and Somewhat, To a high extent, To a very high extent = Yes.) Thereafter, sensitivity and specificity of the limit healthy NFR value were examined in the same way as above. The sensitivity of unhealthy Quantitative demands was 0.733, and of Emotional demands was 0.656. The specificity regarding healthy Quantitative demands was 0.594, and for Emotional demands it was 0.610.

## Discussion

### Easily readable health or risk concerning health and well-being

While the easily readable mental-load reaction NFR from work also represents other decisive factors involved in quality of life, including prospective health and risk concerning health and well-being, there must be a limit healthy NFR value. Therefore, a cut-off value for a healthy NFR value was examined over two years. Since NFR parallels other function impacts^1,19^ such as difficulties concentrating or securing good sleep, the NFR scale of the webNFR was calibrated from relevant impact on functions instruments. This was possible also because these types of effects on health, with relevance for the psychosocial working environment,^33^ have been found to show measured values on the same order of magnitude.^23^

### The importance of the frequency of the load reaction

The NFR score mirrors the frequency of a restrictive load reaction after work, such as feeling exhausted or having problems concentrating. The fatigue at the end of a shift also signals the (lack of) capacity to recover between shifts (from, e.g., rumination about work; see^2^). This scenario implies a risk for convergence into constant fatigue, a transition from a high NFR to, for example, exhaustion and failing ability to recover,^38^ by which is meant one serious disease. The reaction of NFR should at most be irregular and sparse. Therefore, from the calibration to other health scales the NFR limit value both at baseline and after two years was set below regularity, as in “Sometimes.” This corresponds to being below constant recurrence in terms of “A little bit of the time” concerning the other dimensions of health. Logically, compensatory effort also deals with function impacts such as difficulties concentrating.

### Delimited load from work

NFR represents diverse types of load from work,^15,16^ such as lack of Recovery opportunities^1,16^ and even physical workload.^4^ Based on two subgroups (healthy NFR and unhealthy NFR), healthy and unhealthy working conditions were delimited from approximately normal distributions of these conditions and a set level of 0.5 SD from the mean of the two most important conditions. For the two second-most important conditions, these were classified into yes and no categories and delimited in a similar way.

The most important result from calculating the limit values for NFR and the psychosocial work environment is that a limit healthy NFR value was calibrated from health and well-being scales in terms of 0–9 points. From here also follows that 0–9 points on the NFR scale can be used to create healthy limit values for other significant psychosocial working environment factors under other conditions.

### Validity

The predictive sensitivity of the delimited NFR at baseline concerning an unhealthy NFR after two years was tested and found to be 85% (with a specificity of 74%). The concurrent working conditions were tested for sensitivity and specificity regarding an unhealthy or healthy NFR in terms of working conditions that do or don’t jeopardize a healthy NFR. The sensitivity correctly classifying unhealthy load from work ranged from 66% to 75%. Specificity concerning healthy conditions was 54% to 61%.

While the NFR scale represents a load reaction after work, it is distinguishable from milder psychiatric symptoms,^7^ which favors validity (and reliability) of the scale. The instrument further centers on a signal from fatigue, which is more valid for predicting future health status than is sleep disturbance.^23^ As seen in Table 2, and in likeness to the Burn out measurement^42^ NFR seems to be a stable measurement over two years.

### Prevention of accumulation of fatigue during the work shift

Not all work results in NFR,^43^ and therefore, Kästner et al.^2^ saw a need for work redesign, sufﬁcient recovery opportunities, and a reduction of high work intensity that could prevent fatigue accumulation and reduce risks of accidents and health impairment. The implication of the current web instrument is the same.

### Limitations

NFR represents decisive factors involved in the level of health, but the current data do not include neuroendocrinological measurements. Instead, the interpretation of our data from the perspective of correspondence to neuroendocrinological processes relies on the state of knowledge (see Introduction section). In addition, a close correspondence between neuroendocrinological dysregulation and other forms of the restrictive impact on functions from work of decrease in well-being was established as early as the 1970s, for example, fatigue, irritation, and heart rate^44^; psychosomatic symptoms^44,45^; and decreased cognitive function.^46^

The extent to which NFR is explained by working conditions varies between occupations.^1^ The study group represented both academic and non-academic jobs with four diverse sets of occupational characteristics. High NFR was seen in 13% of architects and engineers and in 30% of home care employees.^1^ This difference is likely to be caused by the difference in work demands and recovery opportunities, meaning that gender-typical occupational characteristics are found to lie behind gender differences in work-related risk concerning health and well-being.^47^ The current calculations concerned four diverse sets of occupational characteristics and the associations between work and health of the individuals. However, the participants do not represent occupational populations but instead four very narrowly defined work contexts.

Alternative models to establish limit values for NFR could be to use measures of work ability or labor force exit. In an intervention study using aerobic exercise it was found that after 12 months work ability improved and NFR decreased.^48^ NFR was found to be a precursor to labor force exit.^49^ However, one drawback to using these outcome measures to establish limit values is that they are late consequences of unhealthy NFR. Furthermore, the outcomes of work ability labor force exit are secondary to work demands and the impact on functions of work demands.

The proposed limit values in the current article may be adjusted if, for example, the sensitivity needs to be higher in occupational tasks with high demands of vigilance. A lowered cut-off value would increase the sensitivity but at the same time decrease the specificity.

## Conclusion

A limit healthy NFR value was based on the frequency of the NFR reaction and validated by parallel impact on functions (frequency). The limit values concerning both NFR and psychosocial conditions at work could guide interventions such as improved degrees of freedom concerning breaks and adding resources to facilitate doing good quality work.
